# Two Cases of Curious Sequellas in Rheumatism

**Published:** 1887-04

**Authors:** Thomas C. Osborn

**Affiliations:** Cleburne, Texas, Ex-Vice-President and Ex-President, and Corresponding Member of the Alabama State Medical Association; Member of the American Medical Association; Honorary Member Texas State Medical Association; Ex-President of the Greensbro, Alabama Medical Society; Ex-President of the Lincoln Parish Medical Society; President of the Johnson County Medical Society; Member of the Tennessee Medical Society; Honorary Member of the Atlanta Academy of Medicine; Honorary Member of the Mobile Pathological Society; Author of “A Peculiar Appearance of the Tongue in Malarial Diseases,” &c. &c. &.


### TWO CASES OF CURIOUS SEQUELLÆ IN RHEUMATISM.

*Reported by Thomas C. Osborn, M. D., of Cleburne, Texas,*

Ex-Vice-President and Ex-President, and Corresponding Member of the Alabama State Medical Association; Member of the American Medical Association; Honorary Member Texas State Medical Association; Ex-President of the Greensbro, Alabama Medical Society; Ex-President of the Lincoln Parish Medical Society; President of the Johnson County Medical Society; Member of the Tennessee Medical Society; Honorary Member of the Atlanta Academy of Medicine; Honorary Member of the Mobile Pathological Society; Author of "A Peculiar Appearance of the Tongue in Malarial Diseases," &c. &c. &.

For Daniel's Texas Medical Journal.

"ABSTRACT facts are wanted by medical scientists in erecting and completing the works they are engaged upon."

"They are the proper persons to decide upon the intrinsic value of the points presented to their inspection."

"It is the duty of the workmen—call them hod-carriers, if you choose—to keep them well supplied with suitable materials for the structure they have in hand."

For many years I have been deeply impressed with the truth of the foregoing aphorisms, and having, with the rest of the medical profession, a profound interest in progressive literature, and as my age admonishes me of the night of coming rest, I will no longer delay in reporting the following cases of rheumatism, which, although old to me, have been nevertheless, well preserved in my keeping, and are now tendered to the profession in the hope of serving well—perhaps as key-stones in the corollary arches somewhere in the departments of pathology and physiology, towards both of which they have a decided bearing. The contribution has been held in abeyance, hoping to see in the records other cases of a similar character, but, up to date, I have seen no others in the course of my reading. I do not mean that such instances have never occurred to other observers, but that, if they have, I have failed seeing them in print.

There are few subjects which can be so advantageously discussed in medical journalism as that of rheumatism. Not alone on account of its frequent occurrence, and broadcast character—no latitude, nor longitude escaping its inflictions—but, mainly, because of its erratic nature, and intractable duration under the best methods devised for its management.

It is known to have a decided preference for some portion of the fibrous tissue, especially that in the larger articulations. Its erratic phase is very often observed. Its rapid translation to some one of the vital organs, probably the surroundings of the heart, is sometimes witnessed. Its heredity is so confidently asserted as to make it a factor in matters of life assurance. Its inflamatory nature cannot be questioned; and its resulting deformities can often be safely predicted. But with all this, its intimate pathology is still in doubt; the agent producing it, *materies morbi*, micro-organism, or whatever it is, has not been definitely ascertained; Roberts saying, "in not a few instances no definite cause can be fixed upon, and it is quite conceivable that processes may go on in the system, which gradually tend to generate an amount of the poison sufficient to produce the complaint;" whilst Guttman was unable to find but a few cases in which his staphylococcus aureus seemed to be the cause of the disease. There is no positive proof of its infectiousness. Its sudden translations, differing in this respect from ordinary inflamations, have not been properly accounted for; whilst the vast difference manifested in its disfiguring effects in some cases, and its disorganizing results in others, have not at all been satisfactorily elucidated. Nor has its therapeusis become so well established that physicians can repose much confidence in its successful arrest.

With these prepatory remarks no further reasons are at all necessary in detailing the cases kept so long under consideration.

Case I. Early in February, 1845, in Erie, Greene county, Alabama, a steamboat landing on the Warrior river, I became acquainted with Mr. Abbott, a neighboring planter in Perry county, whose age was 58 years

To casual observers he appeared to be well preserved, but owing to a habit he had of covering his head at all times with a woolen shawl, no one could fail to see that something was seriously wrong with his system. In the course of our conversation, he stated that he was compelled to use the shawl as a protection against the violence of *shocks* which heavy thunder, or other loud noises produced upon his brain; as, when he was not so protected, he would be *stunned*, as from a blow on his head, and *fall* helplessly to the ground.

Being intensely interested in this rare phenomenon, it was but natural with me to wish to know more of his life history, and as he was not unwilling to gratify my curiosity, he made for me the following statement:

At the age of 25 years he suffered acutely with an attack of synovial arthritis in the right knee joint, which, at the end of one week, suddenly ceased paining him, and almost instantly afterward, a violent pain occurred in the top of his head, apparently in the bony tissue, near the intersection of the sagital and lamdoidal sutures, and for the next five days his suffering was as full of agonizing torture as it had been when the knee was affected.

The seat of the suffering appeared to be confined to a small spot, and did not disturb the functions of the brain. There were swelling of the part, and intense tenderness, with a high grade of fever; in fact the fever had continued the same as when the joint was affected. At the end of the fifth day the pain in the head ceased as suddenly as it had done in the knee, and in the meantime, the swelling in the joint had entirely subsided; but all this was immediately followed by an insatiable thirst, requiring for its alleviation copious draughts of water frequently repeated. In the course of an hour later his kidneys assumed extraordinary activity, and the flow of urine was at all times equal to the water consumed; and this condition of things was kept up persistently the rest of his life, or, more correctly speaking, until his death in 1859. There was neither anchylosis nor deformity left in the knee joint, nor were his mental faculties at any time disturbed or seriously clouded, only in brooding anxieties as to the next phase of the erratic disease.

In the course of two months from the date of his thirst, there appeared, at the spot in the cranium where the pain had been seated, a small soft spot, which continued enlarging in every direction, until at the time I saw him all the parietalia, the os occipitis as far down as its protuberence, and the os frontis on a line with the supercilliary ridges, were totally absorbed, leaving only the squamous portions of temporal bones to interrupt the regular curvature of the osseous line around the calvarium—the bony arch had entirely disappeared—and there was nothing intervening between the dura mater and the superincumbent muscular and tegumentary tissues, over which there still remained a profusion of dark-colored hair.

For thirty years, or more, this disintegration of bone had been relentlessly progressing, *without otherwise apparently disturbing any other parts of his organism!* There was not only his assurance that he consumed three gallons of water every 24 hours, with an equal quantity of urinary flow, but I was likewise cognizant of the facts. We took a three gallon bucket of water and placed it beside his bed at 8 o'clock p. m., and set another bucket of the same size in the room to catch the urine; and at 8 o'clock p. m. the next day, the water-bucket was empty, whilst the other bucket was full of urine.

An unvarying custom of Mr. Abbott was to lie down, or be seated on the approach of thunder storms, as a protection against the sudden shocks and falls which would result, and this habit had been adopted after several sad experiences.

He had a family of children, some of whom were grown, and one ot these accompanied him whenever he went traveling. Every year he made a trip by steamboat to Mobile to see after the sales of his cotton crops, and to purchase necessary supplies for family and plantation uses. Those were days when commission merchants controlled the entire selling and purchasing business for nearly everybody in the country for hundreds of miles away. But now all this is *radically* changed, and we thank the Radicals for it.

In reviewing this strange freak of rheumatism, may we not conceive that the several translations in its course were, first from the synovial tissue of the knee-joint, to the diploe of the cranial bones; and next, from the diplœ to the tubuli uriniferi of one or both kidneys? Or, as he had never suffered with much distress in the lumbar regions, should we conclude that the activity of the renal organs was, in a great measure, compensatory in function, to offset the enormous quantity of water consumed? In the latter supposition it would seem necessary to infer that the second translation was directed to the absorbent system; in which case there would, of course, be more or less emaciation of the whole body. But as he was well preserved in body, save only the osseous disintegration, we are left to conclude that the thirst was due to the preternatural activity of the kidneys.

Aside from either hypothesis, I am impressed with the conviction that cases of translations of rheumatism are only seen where the original site *is in the synovial tissue*; and many cases subsequent to this one have served to confirm the opinion.

It is, I think, equally certain that when the attack is made on any one portion of the endo-ostium, there will in all probability be more or less erratic translation observed, and the result will be, aside from pericardial determination, either mollities ossium from inordinate absorption of bone, or an imperfect secretion of phosphate of lime, or in defective ossification; or of absorption of medullary tissue, constituting brittle bone; or, if connected with a syphilitic taint, a resulting sarcoma in, or near the part affected; for the simple reason that the endo-ostium is an absorbing tissue, and there can hardly be a doubt that in diplœ there is an abundance of endo-ostium involved.

In other, and terser language, the synovial and endo-ostial tissues belong to the same system, and serve parallel purposes.

On the other hand, it is equally probable that when the seat of rheumatism is confined to the periostium, the resulting evils will be in the form of exostoses; or nodosities; or caries and suppuration, or exfoliation; or anchylosis and distortion of the joints.

One other proposition has forcibly impressed itself upon my mind, which has not been seen by me in any treatise on rheumatism, and that is, where, after an acute inflamatory attack of the disease, there is any one of the results mentioned in the foregoing paragraphs, there is *never a subsequent attack of the inflamatory form* in the same individual, but an almost continuous form of the chronic variety, in which the muscular or tendonous systems seem to have an inordinate share.

Case II. On the plantation belonging to the late Col. Samuel Pickens, known as his "Cane-brake place," in Hale county, Alabama, whilst on my rounds in the quarter visiting sick negroes in the year 1859, it was proposed by Mr. I. S. High, the intelligent and humane superintendent, that I should go into the nursery-room, which we were at the moment passing, and see what he called a pathological curiosity.

The subject was a small-sized negro man, and was introduced to me as Lewis, aged 36 years, who had been, for the last 20 years, prone and stiff and straight upon his bed, with anchylosis of every joint in his body, except the maxillary articulations, and a very slight lateral motion in the right wrist-joint. He was quite helpless, and rigid as a log of wood. His legs were straight and fixed close to each other, and there was no contraction of either feet or toes. The left arm rested diagonally across his abdomen, with the cramped hand and fingers settled near the right illiac region. The right arm was bent across the chest, the thumb and index finger nearly straight and closely parallel, whilst the three remaining fingers were tightly drawn upon the palm—the hand resting on the left nipple; and with a wooden padle ten inches long placed as occasion required between the thumb and finger, and from a plate or pan of food resting on his left arm and chest, he could and prefered feeding himself at meal times.

Lifting him at the head or feet was as raising a log on end. And yet this man, in this condition, claimed that he was not a totally useless being in that busy hive of negroes ! His lower jaw had its natural motion; his eyes sparkled with intelligence, and his tongue and lips tripped glibly over words in sprightly conversation. His memory was tenacious. His voice was soft and easily modulated, and he was at all times ready to take a part in talking with cheerful visitors, especially on subjects pertaining to religion, or in reference to himself. One of his vain points was in repeating from memory alone, verse about, with any person, in the Bible; bantering the reader to skip anywhere in the volume, read any verse he or she might select, and he would repeat the next one above or below it, if it was wished, and give the book, chapter, and number of the verses recited. All this he had learned by ear, and did not know a letter by sight in the alphabet. He seemed proud of his ability to be of service in the house, and, in proof of this, he claimed the privilege of rocking all the cradles in the long nursery when the mothers of the babies were absent at work in the fields. To do this was an amusement as well as pleasure to him; and he also prided himself in telling the age of every child that had been born on the quarter since his confinement.

The method adopted for rocking the cradles was an invention of his own, and consisted in lashing the cradles in a row, side by side, and with a leading string from the nearest fixed between his thumb and finger, the little lateral motion of the wrist was just sufficient to set the whole number in gentle agitation.

He was indeed a great curiosity to me. So much so that I grew deeply interested in the following history of his affliction; and now to the scientist the really strangest part of this phenominal being is yet to be given.

At the age of 16 years he was attacked with inflammatory rheumatism in the right knee and leg; then in the right shoulder and arm; then in left limbs successively, until his entire body was symmetrically afflicted with the disease, suffering excruciating agonies, accompanied with a high grade of fever, and considerable emaciation. This condition lasted for three months or longer, and terminated in progressive anchylosis of all his joints, except those already designated. His *growth* was arrested, but his mental faculties, his appetite and digestion, thirst, and daily defecations, were left in an unmolested condition during the remaining years of his life. His respiration, and the movements of his heart were normal, but the respiration was exclusively abdominal, the chest being as fixed as a metallic box.

On exposing his body, I found the skin ashy and harsh everywhere except the abdomen and face. The muscles were hard, and considerably shrunken; the bones were slightly enlarged, the ribs stationary, and there was no softness found except on the abdomen and face—the abdomen neither shrunken nor full. He had not, since his confinement, perspired, only on the soft parts already noticed.

At short distances apart on his legs, back, arms, and thorax, there were many nodosities, in sizes varying from a buck-shot to that of a medium orange, the largest and greatest abundance being located upon the legs, and next on the shoulders and arms—the nodes giving to my hand the feeling of bone. On the chest and head they were numerous, but smaller. The superficial veins, the cephalic vein and the radial and ulnar arteries, were like bony ridges, in some places enlarged to the size of a man's thumb, and on the limbs, hands and feet, the muscles were like so many hard pieces of wood. It was left to conjecture whether the vessels conveyed blood to and from the heart, for such a thing as feeling the current waves in either artery or vein was entirely obscured. The functions of the kidneys were normal, and the genital organs had escaped any part in the infliction. He had never been tainted with venereal infection, nor did ne manifest an ordinary share of sensual propensity.

This man was alive in the beginning ot the year 1865, but I lost sight of him after the termination of the war.

In submitting this report to those medical scientists for whom it is prepared, I claim having only attempted portraying the cases with artistic fidelity, irrespective of scenic drapery or relief, except such as I thought necessary to attract a favorable interest in the striking peculiarities of the sequellæ; and therefore, the mechanical execution of the pictures may be open to justifiable criticism. Be that as it may, I will still retain a conscientious satisfaction in the reality of having performed a sacred duty to the profession of medicine, even though I am liable to the censure of having unnecessarily delayed its performance for so great a length of time.

